# Inhibition of *Escherichia coli* chromosome replication by rifampicin treatment or during the stringent response is overcome by de novo DnaA protein synthesis

**DOI:** 10.1111/mmi.14531

**Published:** 2020-06-15

**Authors:** Leise Riber, Anders Løbner‐Olesen

**Affiliations:** ^1^ Department of Biology University of Copenhagen Copenhagen Denmark

**Keywords:** DnaA, *Escherichia coli*, ppGpp, replication initiation, rifampicin, stringent response, transcriptional activation

## Abstract

Initiation of *Escherichia coli* chromosome replication is controlled by the DnaA initiator protein. Both rifampicin‐mediated inhibition of transcription and ppGpp‐induced changes in global transcription stops replication at the level of initiation. Here, we show that continued DnaA protein synthesis allows for replication initiation both during the rifampicin treatment and during the stringent response when the ppGpp level is high. A reduction in or cessation of de novo DnaA synthesis, therefore, causes the initiation arrest in both cases. In accordance with this, inhibition of translation with chloramphenicol also stops initiations. The initiation arrest caused by rifampicin was faster than that caused by chloramphenicol, despite of the latter inhibiting DnaA accumulation immediately. During chloramphenicol treatment transcription is still ongoing and we suggest that transcriptional events in or near the origin, that is, transcriptional activation, can allow for a few extra initiations when DnaA becomes limiting. We suggest, for both rifampicin treated cells and for cells accumulating ppGpp, that a turn‐off of initiation from *oriC* requires a stop in de novo DnaA synthesis and that an additional lack of transcriptional activation enhances this process, that is, leads to a faster initiation stop.

## Graphical abstract

Both rifampicin and high levels of ppGpp affect the *E. coli* RNA polymerase (RNAP), and lead to reduced *dnaA* gene transcription and cessation of initiation of chromosome replication (a). Replication initiation does however continue when DnaA is de novo synthesized from a rifampicin‐resistant and ppGpp‐insensitive T7 RNAP‐dependent promoter (b). Therefore, both rifampicin and ppGpp inhibit initiation of replication primarily by limiting DnaA synthesis.

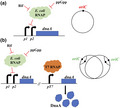

## 1 INTRODUCTION

In *Escherichia coli* chromosome replication commences from a single origin of replication, *oriC*. The *oriC* region contains an AT‐rich region as well as multiple binding sites for the DnaA initiator protein (Leonard and Mechali, 2013). The DnaA protein belongs to the family of AAA^+^ (ATPases associated with diverse cellular activities) proteins. DnaA binds ATP and ADP with similar affinities (Sekimizu *et al*., 1987), but only the ATP‐bound form, DnaA^ATP^, is active in replication initiation (Skarstad and Katayama, 2013). At initiation DnaA^ATP^ oligomerizes on *oriC* in a highly ordered manner, which leads to origin unwinding in the AT‐rich region, and subsequent loading of two replisomes to direct chromosome replication in a bidirectional manner, until terminated at the opposite replication terminus region, *terC*.

Initiation of chromosome replication is a highly regulated step in the *E. coli* cell cycle during which all cellular origins are simultaneously initiated once and only once per cell cycle (Boye *et al*., 2000). This tight control is ensured mainly by fluctuations in the cellular DnaA^ATP^‐to‐DnaA^ADP^ ratio throughout the cell cycle, in which an increase to a certain threshold will set the time of initiation (reviewed by Skarstad and Katayama, 2013; Riber *et al*., 2016). Following initiation, two processes convert active DnaA^ATP^ into inactive DnaA^ADP^. First, RIDA (Regulatory Inactivation of DnaA) stimulates the intrinsic ATPase activity of DnaA through the action of the Hda protein in association with the DNA‐loaded β‐clamp (Katayama *et al*., 1998; Kato and Katayama, 2001). Second, DDAH (*datA*‐dependent DnaA^ATP^ hydrolysis) stimulates DnaA^ATP^ hydrolysis at the *datA* locus (Kasho and Katayama, 2013). Later in the cell cycle, rejuvenation of DnaA at the *DARS1+2* regions facilitates an increase in the DnaA^ATP^‐to‐DnaA^ADP^ ratio (Fujimitsu *et al*., 2009).

The broad‐spectrum antibiotic rifampicin inhibits initiation of global RNA synthesis by high‐affinity binding to the bacterial DNA‐dependent RNA polymerase, RNAP (Hartmann *et al*., 1967). Rifampicin has been known to inhibit replication initiation for decades (von Meyenburg *et al*., 1979), while having no effect on replication elongation. This feature is often employed for determination of the number of replication origins per cell by flow cytometry (Skarstad *et al*., 1986). The exact mechanisms by which rifampicin inhibits replication initiation remain unclear, but two models are frequently proposed. In one suggestion, a consequence of transcription inhibition is that de novo DnaA protein synthesis ceases, and hence initiation is inhibited. Inhibition of protein synthesis by chloramphenicol (Vazquez, 1979) is in agreement with this hypothesis. Another suggestion is that transcriptional activation of *oriC* is required for replication initiation and this process is inhibited by rifampicin. Specifically, transcriptional activity of promoters located with close proximity to or within *oriC* is known to alter the DNA structure in such a way that facilitates DNA duplex unwinding, either by creating a stable R‐loop, or by introducing negative supercoils in the wake of the migrating RNA polymerase complex, thereby stimulating replication initiation (Magnan and Bates, 2015). Transcription units located close to *oriC* include promoters of the highly conserved *mioC* and *gidA* genes. Given their opposite orientations, *mioC* transcripts read toward *oriC* introducing initiation‐inhibiting positive supercoils, whereas *gidA* transcripts read away from *oriC* introducing initiation‐activating negative supercoils (Theisen *et al*., 1993; Bates *et al*., 1997). Some *mioC* transcripts will progress completely through *oriC* (Rokeach and Zyskind, 1986).

Initiation of chromosome replication is also inhibited by accumulation of the secondary messenger guanosine tetra‐ or penta‐phosphate, ppGpp, and pppGpp, respectively (together abbreviated as ppGpp below (Levine *et al*., 1991, Schreiber *et al*., 1995, Ferullo and Lovett, 2008)). However, as with rifampicin, the exact mechanism by which ppGpp accumulation contributes to this inhibition is not well understood. ppGpp accumulates during stressful conditions, such as amino acid starvation, iron limitation, heat shock, and antibiotic treatment in what is collectively referred to as the “stringent response” (Dalebroux and Swanson, 2012; Hauryliuk *et al*., 2015). Two enzymes, RelA and SpoT, control the levels of ppGpp. The main ppGpp synthetase, RelA, synthesizes ppGpp when associating with ribosomes in the ribosomal acceptor site (A‐site) to sense uncharged tRNAs during amino acid starvation (Cashel *et al*., 1996; Potrykus and Cashel, 2008; Hauryliuk *et al*., 2015). SpoT, moreover, exhibits dual functions and is able to synthesize ppGpp in response to starvation of other nutrients, such as iron, fatty acids, and certain carbon sources, as well as being responsible for hydrolyzing ppGpp and resetting cells after ppGpp induction (Sarubbi *et al*., 1989). Activation of the stringent response causes significant alterations in global gene expression, which are driven by the direct binding of ppGpp to two sites in the bacterial RNAP located at the interface of the ω and β’ subunits (Ross *et al*., 2013; 2016). Binding of ppGpp to RNAP results in an extensive transcriptional reprograming including downregulation of genes involved in stable RNA (ribosomal RNA; rRNA, and transfer RNA; tRNA, respectively), synthesis, DNA replication, transcription, translation, and various metabolic pathways, while genes related to stress and amino acid biosynthesis are upregulated (Cashel *et al*., 1996; Haugen *et al*., 2008; Potrykus and Cashel, 2008; Traxler *et al*., 2008; Kanjee *et al*., 2012; Hauryliuk *et al*., 2015). Overall, these transcriptional changes assist bacteria in entering a physiologically state that enable them to survive the stressful conditions.

The effects of ppGpp on replication initiation is argued to be indirect, that is, a result of changes in general cell physiology and rate of stable RNA synthesis (Hernandez and Bremer, 1993). However, *dnaA* is among the genes downregulated by ppGpp with the transcriptional activity of both *dnaA* gene promoters, *dnaAp1* and *dnaAp2* (Hansen *et al*., 1982), being reduced in a RelA‐dependent manner (Chiaramello and Zyskind, 1990; Zyskind and Smith, 1992). Lack of de novo DnaA synthesis or even degradation (Gross and Konieczny, 2020) during ppGpp accumulation could therefore contribute to, or even be responsible for, the cessation of initiation seen in the presence of ppGpp. A recent study further suggests that a ppGpp‐driven reduction in transcripts from promoters located close to *oriC*, leading to less negative supercoiling of *oriC*, that is, less transcriptional activation, could explain the negative effect of ppGpp on replication initiation (Kraemer *et al*., 2019).

Here, we address the role of DnaA limitation in rifampicin‐ and ppGpp‐mediated inhibition of DNA replication initiation. We show that driving *dnaA* gene transcription continuously from a rifampicin‐resistant and ppGpp‐insensitive T7 RNAP‐dependent promoter, p_T7_, enables cells to continue replication initiation in the presence of both rifampicin and RelA‐synthesized ppGpp.

## 2 RESULTS

### 2.1 Continuous synthesis of DnaA allows initiation of chromosome replication in the presence of rifampicin

**FIGURE 1:**
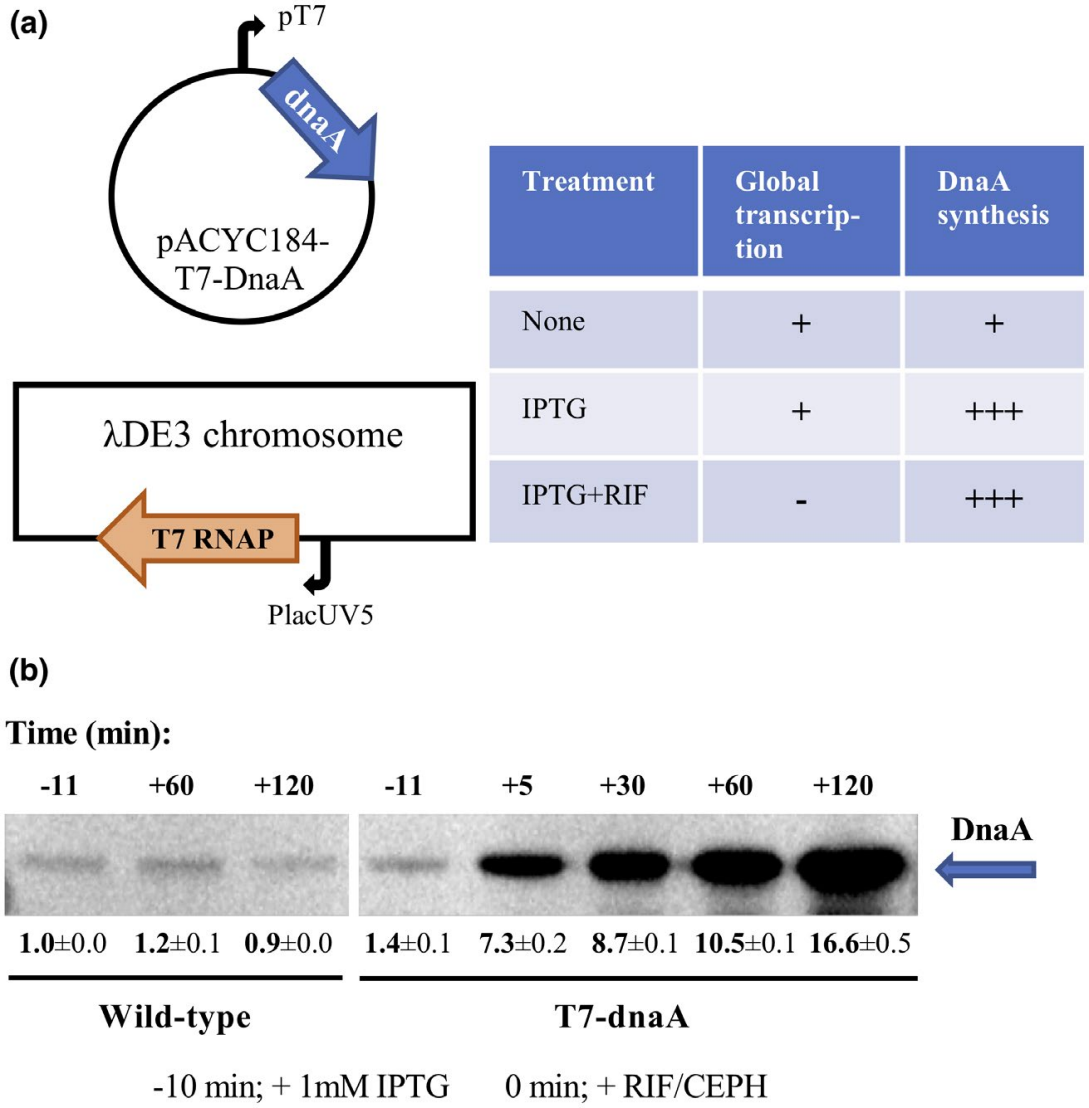
DnaA is synthesized from a T7 RNAP‐dependent promoter during rifampicin treatment. (a) Schematic overview of the applied molecular system. The bacteriophage T7 RNA polymerase (T7 RNAP) is resistant to rifampicin (Tabor, 2001). IPTG‐mediated induction of the *lac*UV5 promoter leads to expression of the chromosomally encoded T7 RNAP, which induce transcription of *dnaA* from promoter p_T7_. During rifampicin treatment, global RNA synthesis initiation is blocked, whereas the *dnaA* gene is continuously expressed under the control of p_T7_ in the presence of IPTG. (b) DnaA protein content determined by Western blot analysis for cells carrying the “empty” pACYC184‐T7 plasmid (Wild type; left) or the DnaA expression plasmid, pACYC184‐T7‐DnaA (T7‐dnaA; right), at the indicated times. Cells were grown at 37°C in AB minimal medium supplemented with glucose and casamino acids. At time −10 min 1 mM IPTG was added to induce T7 RNAP synthesis, and at time 0 min rifampicin and cephalexin was added to block initiation of RNA synthesis and cell division, respectively. Samples were adjusted to the same total protein content before loading onto the Western blot. All quantifications, normalized to pretreated wild‐type cells (−11 min), include standard deviations and are based on triplicates.

**FIGURE 2:**
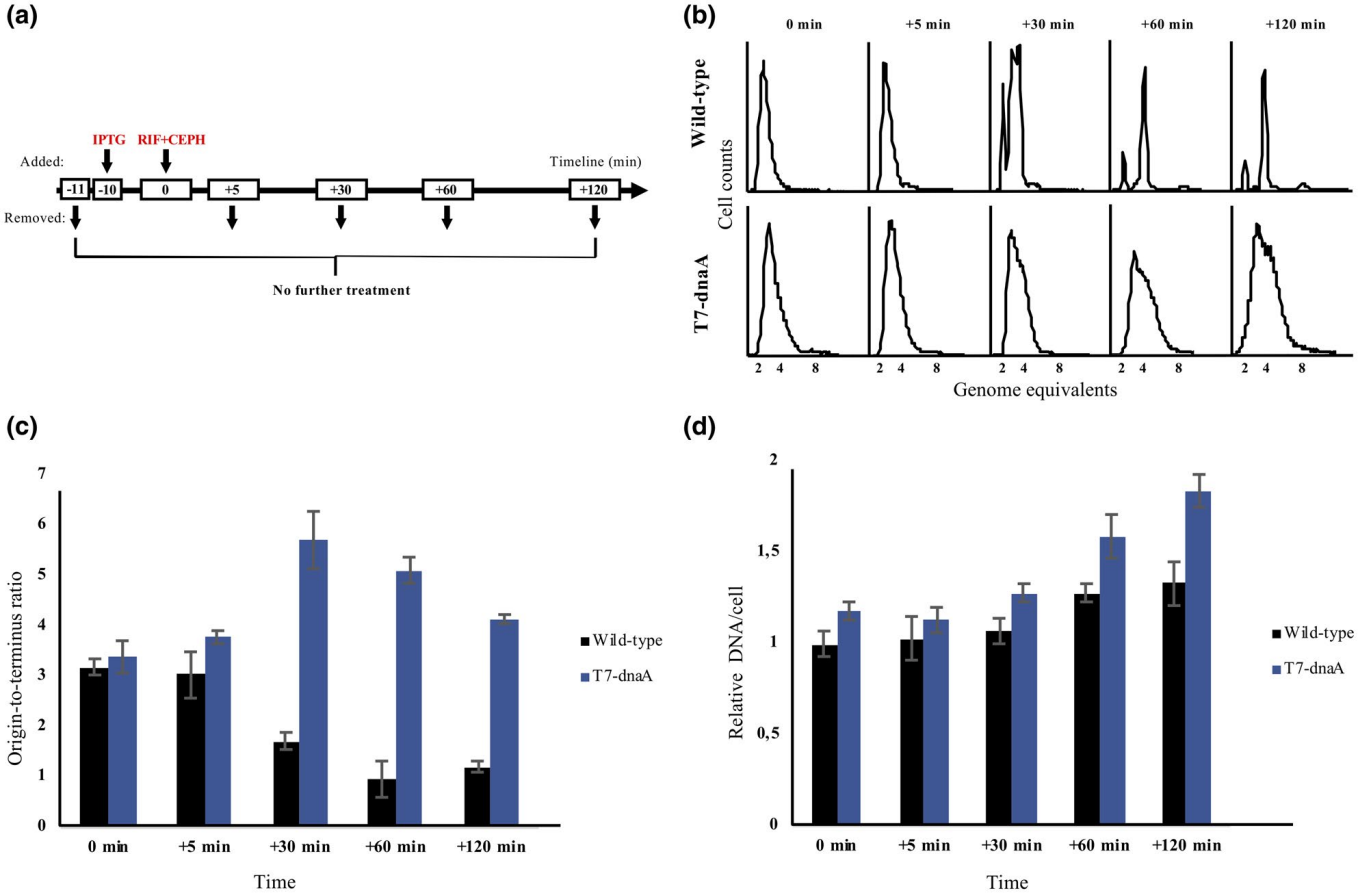
de novo DnaA synthesis allows replication initiation in the presence of rifampicin. (a) Overview of the timeline of the experiment. (b) Cells of strain ALO6511 carrying the “empty” pACYC184‐T7 plasmid (Wild type; upper panel) or the DnaA expression plasmid, pACYC184‐T7‐DnaA (T7‐dnaA; lower panel), were grown at 37°C in AB minimal medium supplemented with glucose and casamino acids. At time −10 min 1 mM IPTG was added to induce T7 RNAP and hence DnaA synthesis, and at time 0 min rifampicin and cephalexin was added to block initiation of RNA synthesis and cell division, respectively. Samples for flow cytometry analysis and quantitative PCR (qPCR) were removed at the indicated timepoints. (c) Origin‐to‐terminus ratios for cells of strain ALO6511 carrying the “empty” pACYC184‐T7 plasmid (Wild type) or the DnaA expression plasmid, pACYC184‐T7‐DnaA (T7‐dnaA) at the indicated time points. (d) Cellular DNA content (DNA/cell) for cells of strain ALO6511 carrying the “empty” pACYC184‐T7 plasmid (Wild type) or pACYC184‐T7‐DnaA (T7‐dnaA) at the indicated time points. DNA/cell is normalized to wild‐type cells at *t* = 0 min (the time of rifampicin and cephalexin addition). Standard deviations are based on duplicates.

To determine whether rifampicin blocks replication initiation indirectly through a stop in de novo DnaA protein synthesis, we constructed a MG1655 based strain where DnaA is produced even if rifampicin is present. Briefly, this strain carried a chromosomal λDE3 with the rifampicin‐resistant bacteriophage T7 RNAP under control of the isopropyl β‐D‐1‐thiogalactopyranoside (IPTG) inducible p*lac*UV5 promoter (Tseng *et al*., 2009), and a plasmid, pACYC184‐T7‐DnaA, containing the *dnaA* gene under the control of a T7 RNAP‐dependent promoter, p_T7_ (Tabor, 2001) (Figure 1a). Cells were treated with IPTG for 10 min to allow for synthesis of T7 RNAP prior to addition of rifampicin and cephalexin (Figure 2a). Because rifampicin blocks *E. coli* RNAP, but not the T7 RNAP, DnaA was the sole protein synthesized during rifampicin treatment and it accumulated approximately 16‐fold over 2 hr (Figure 1b). Cephalexin prevented residual cell division during rifampicin treatment.

Wild‐type cells treated with rifampicin and cephalexin ceased initiations while replication elongation continued. Consequently they ended up containing integral numbers of fully replicated chromosomes over time (Figure 2b). Also, the origin‐to‐terminus (*ori*/*ter*) ratio, reflecting the copy number ratio of *oriC* to *terC*, approached 1 over time indicating that cells had finished on‐going replication and carried a complete set of fully replicated chromosome(s), also defined as run out replication (Figure 2c; black bars). The cellular DNA content increased somewhat as a result of run out replication (Figure 2d; black bars). No decrease in DnaA protein level was observed in agreement with the DnaA protein being stable (Torheim *et al*., 2000). Cells that continued to express DnaA in the presence of rifampicin and cephalexin behaved quite different. No clear run out replication phenotype was observed (Figure 2b), the *ori*/*ter* ratio increased after drug addition, representing cells with on‐going chromosome replication (Figure 2c; blue bars), and the cellular DNA content continued to increase for 120 min, which was the duration of the experiment (Figure 2d; blue bars).

These data show that continuous de novo synthesis of DnaA is sufficient to initiate new rounds of DNA replication in the presence of rifampicin. As rifampicin blocks initiation of global RNA synthesis, including transcription from promoters located in or around *oriC,* transcriptional activation of the origin is not necessary for initiation to take place under these conditions. This suggests that rifampicin inhibits initiation of DNA replication primarily through preventing de novo DnaA protein synthesis.

### 2.2 Continuous synthesis of DnaA allows initiation of chromosome replication in the presence of ppGpp

For decades, initiation of chromosome replication has been known to be inhibited by accumulation of ppGpp during the stringent response (Levine *et al*., 1991; Schreiber *et al*., 1995; Ferullo and Lovett, 2008). *dnaA* is downregulated by ppGpp (Chiaramello and Zyskind, 1990), thus it was investigated whether accumulation of ppGpp inhibits replication initiation by preventing de novo DnaA protein synthesis, or by preventing transcriptional activation of *oriC* as previously suggested (Kraemer *et al*., 2019), or both.

**FIGURE 3:**
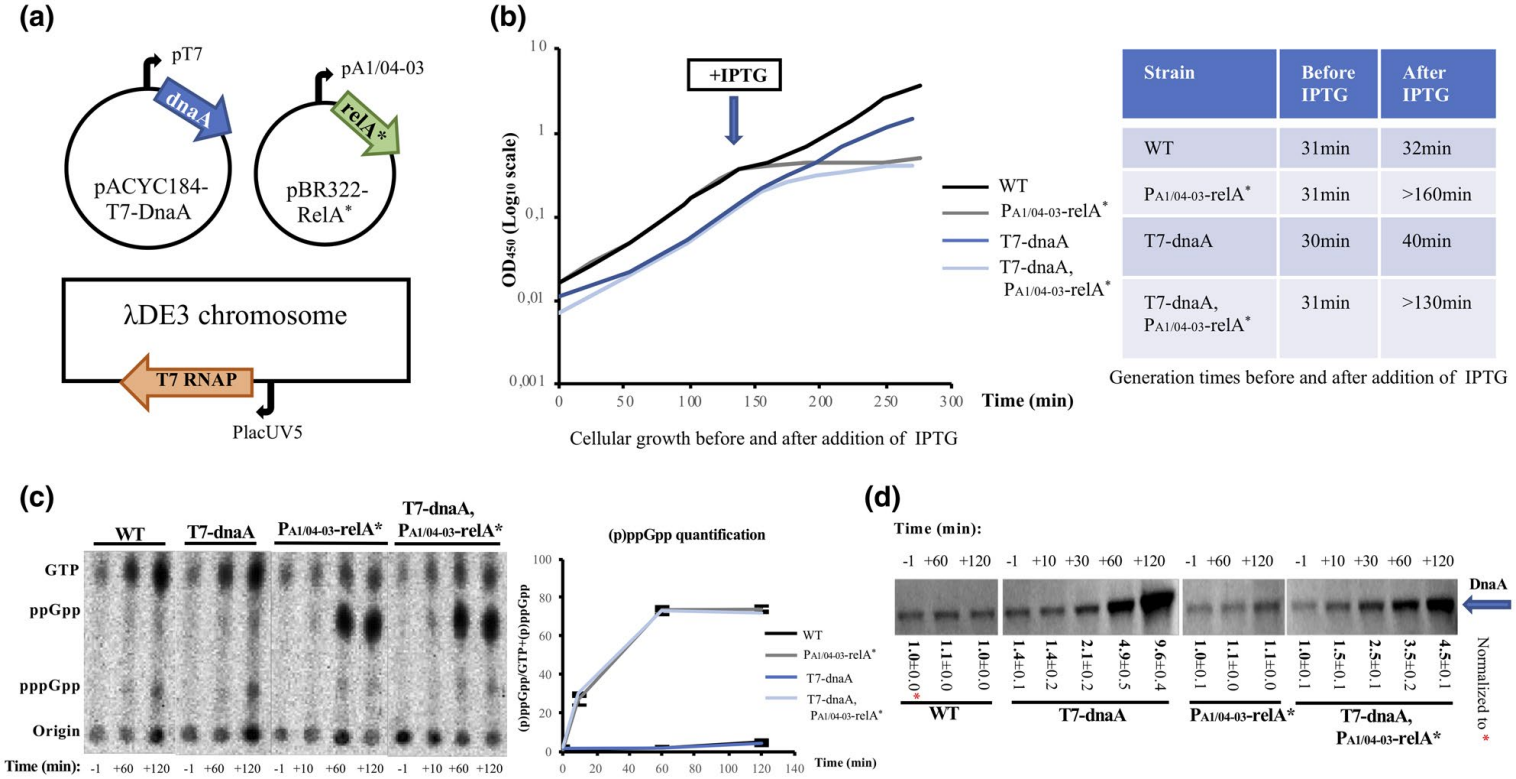
DnaA is synthesized from a T7 RNAP‐dependent promoter in the presence of ppGpp. (a) Overview of the applied molecular system. The *dnaA* gene is under the control of the rifampicin‐resistant and ppGpp‐insensitive bacteriophage T7 RNAP‐dependent promoter, p_T7_, on a pACYC184 derived plasmid. The relA^*^ gene, carried by a pBR322 derivative, is expressed from the p_A1/04‐03_ promoter (Lanzer and Bujard, 1988), which leads to the parallel accumulation of DnaA and ppGpp in the presence of IPTG. See also description for Figure 1a. (b) Growth curves for cells of strain ALO6511 carrying the “empty” plasmid, pACYC184‐T7 and the “control” plasmid, pBR322‐RelA^–^ (WT; black), or pACYC184‐T7 and the RelA expression plasmid, pBR322‐RelA^*^ (P_A1/04‐03_‐relA^*^; gray), and for cells of strain ALO6511 carrying the DnaA expression plasmid, pACYC184‐T7‐DnaA and the “control” plasmid, pBR322‐RelA^–^ (T7‐dnaA; dark blue), or pACYC184‐T7‐DnaA and the RelA expression plasmid, pBR322‐RelA^*^ (T7‐dnaA, P_A1/04‐03_‐relA^*^; light blue). Time of addition of 1 mM IPTG is indicated. Generation times before and after IPTG addition are indicated in the table. (c) Quantification of (p)ppGpp accumulation (total amount of ppGpp and pppGpp) in ALO6511 cells carrying the “empty” plasmid, pACYC184‐T7 and the “control” plasmid, pBR322‐RelA^–^ (WT), pACYC184‐T7 and the RelA expression plasmid, pBR322‐RelA^*^ (P_A1/04‐03_‐relA^*^), and for ALO6511 cells carrying the DnaA expression plasmid, pACYC184‐T7‐DnaA and the “control” plasmid, pBR322‐RelA^–^ (T7‐dnaA), or pACYC184‐T7‐DnaA and the RelA expression plasmid, pBR322‐RelA^*^ (T7‐dnaA, P_A1/04‐03_‐relA^*^), at the indicated times. Autoradiograms (left) were used to calculate and visualize the fractional content of (p)ppGpp as a function of time. (d) DnaA protein content determined by Western blot analysis for ALO6511 cells carrying the “empty” plasmid, pACYC184‐T7 and the “control” plasmid, pBR322‐RelA^–^ (WT), pACYC184‐T7 and the RelA expression plasmid, pBR322‐RelA^*^ (P_A1/04‐03_‐relA^*^), and for ALO6511 cells carrying the DnaA expression plasmid, pACYC184‐T7‐DnaA and the “control” plasmid, pBR322‐RelA^–^ (T7‐dnaA), or pACYC184‐T7‐DnaA and the RelA expression plasmid, pBR322‐RelA^*^ (T7‐dnaA, P_A1/04‐03_‐relA^*^), at the indicated times. Samples were adjusted to the same total protein content before loading onto the Western blot. All quantifications were normalized to pretreated wild‐type cells (−1 min). Standard deviations are based on triplicates.

To address this question, we used the pACYC184‐T7‐DnaA expression plasmid described above and constructed a compatible plasmid, pBR322‐RelA^*^, where the active 455 amino acid N‐terminal *relA* fragment (Svitil *et al*., 1993) is controlled by the IPTG inducible p_A1/04‐03_ promoter (Lanzer and Bujard, 1988). The use of these two plasmids alone or together in the MG1655 λDE3 strain allows for overproduction of DnaA, ppGpp, or both in the same cells in an IPTG dependent manner (Figure 3a). The simultaneous production of ppGpp and DnaA was possible as the *dnaA* gene is transcribed from the ppGpp‐insensitive T7 RNAP‐dependent promoter, p_T7_ (Friesen and Fiil, 1973; Tabor, 2001). This allowed us to study the effect of on‐going DnaA synthesis on replication initiation in the presence of ppGpp.

Induction of RelA^*^ either alone or together with DnaA slowed down mass accumulation (Figure 3b), most likely as a result of increasing ppGpp levels (Figure 3c). DnaA accumulation alone did not affect growth to any significant level, at least not within the timeframe of the experiment (Figure 3b). DnaA levels increased approximately 10‐fold and 5‐fold following IPTG induction in the absence and presence of simultaneous ppGpp accumulation, respectively (Figure 3d). The smaller increase in DnaA level seen in the presence of high ppGpp may result from a reduction in transcription from the native chromosomal copy of the *dnaA* gene (Chiaramello and Zyskind, 1990) and/or from a decrease in DnaA protein translation rate caused by ppGpp‐mediated inhibition of promoters for rRNA and tRNA synthesis (Potrykus and Cashel, 2008). We find it unlikely that ppGpp‐induced degradation of DnaA (Gross and Konieczny, 2020) affected its accumulation as we observed no decrease in DnaA level when ppGpp was increased in otherwise wild‐type cells (Figure 3d).

**FIGURE 4:**
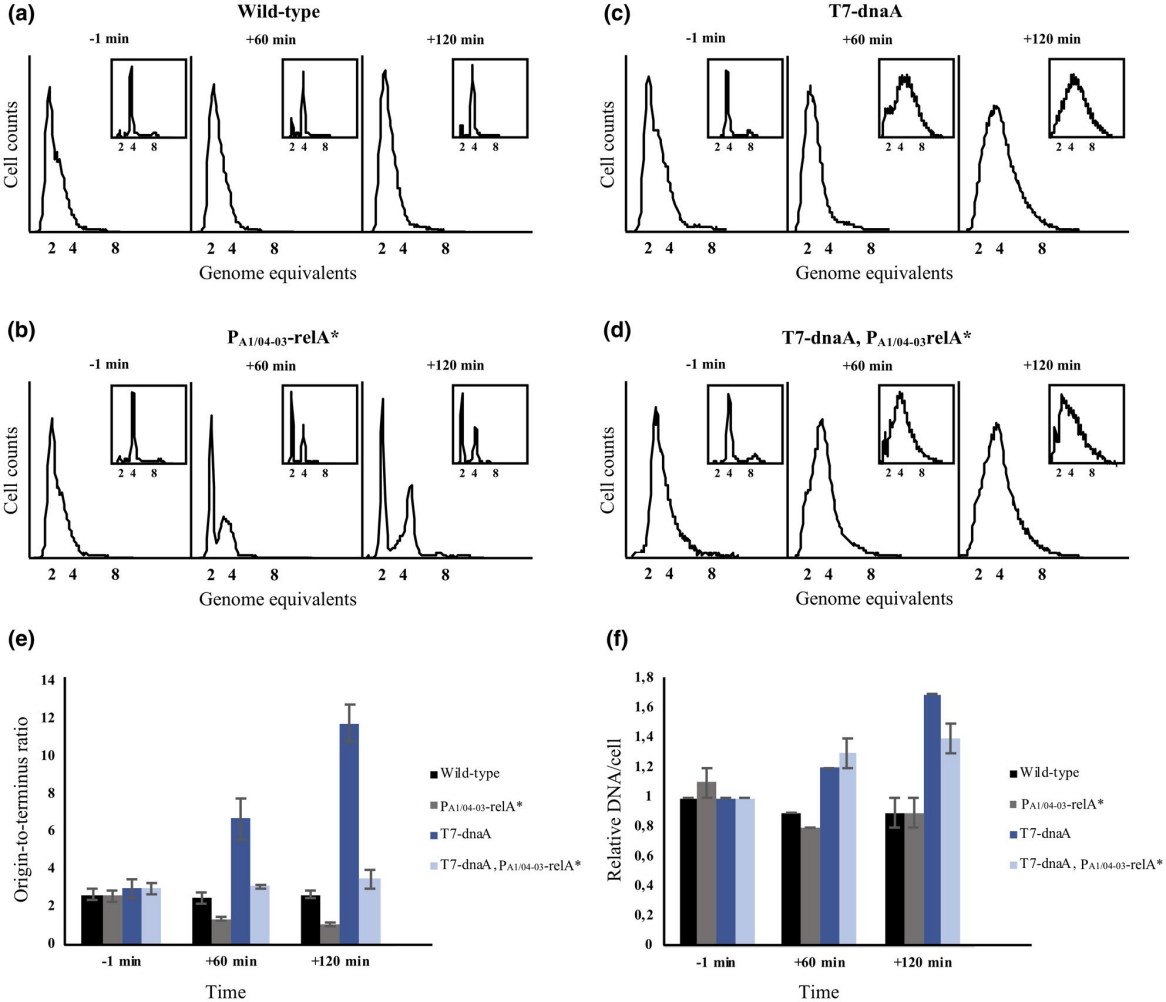
de novo DnaA synthesis allows replication initiation in the presence of ppGpp. (a) Cells of strain ALO6511 carrying the “empty” plasmid, pACYC184‐T7 and the “control” plasmid, pBR322‐RelA^–^ (Wild type), (b) pACYC184‐T7 and the RelA expression plasmid, pBR322‐RelA^*^ (P_A1/04‐03_‐relA^*^), (c) the DnaA expression plasmid, pACYC184‐T7‐DnaA and the “control” plasmid, pBR322‐RelA^–^ (T7‐dnaA), or (d) pACYC184‐T7‐DnaA and the RelA expression plasmid, pBR322‐RelA^*^ (T7‐dnaA, P_A1/04‐03_‐relA^*^). Cells were grown at 37°C in AB minimal medium supplemented with glucose and casamino acids. At time 0 min 1 mM IPTG was added and samples of exponentially growing cells were removed at the indicated times for analysis by flow cytometry and quantitative PCR (qPCR) as well as for treatment with rifampicin and cephalexin for 4 hr prior to flow cytometry analysis (inserts). (e) Origin‐to‐terminus ratios for exponentially growing cells (“Wild‐type,” “P_A1/04‐03_‐relA,^*^” “T7‐dnaA,” and “T7‐dnaA, P_A1/04‐03_‐relA^*^,” respectively), at the indicated times. (f) Cellular DNA content (DNA/cell) for exponentially growing cells (“Wild‐type,” “P_A1/04‐03_‐relA^*^,” “T7‐dnaA,” and “T7‐dnaA, P_A1/04‐03_‐relA^*^,” respectively), at the indicated times. DNA/cell is normalized to wild‐type cells at *t* = −1 min. Standard deviations are based on duplicates.

When analyzed by flow cytometry, cells overproducing ppGpp resembled rifampicin treated wild‐type cells in that replication initiation rapidly ceased so that cells contained fully replicated chromosomes (compare Figure 4a to b). Treatment with rifampicin and cephalexin resulted in wild‐type cells having two or mainly four fully replicated chromosomes as compared to cells accumulating ppGpp where cells had mainly two and some four fully replicated chromosomes, suggesting some residual cell division in the latter (Figure 4a,b; inserts). In both cases the *ori/ter* ratios approached 1 (Figure 4e; gray bar). This is in agreement with previous observations (Ferullo and Lovett, 2008; Kraemer *et al*., 2019). The replication elongation rate did not seem significantly affected within the timeframe of the experiment, as also seen in earlier studies (Levine *et al*., 1991; Ferullo and Lovett, 2008; Denapoli *et al*., 2013). Moreover, overproduction of DnaA resulted in an increase in cellular origin content along with an inability to finish replication in the presence of rifampicin (Figure 4c). Both the *ori/ter* ratios increased dramatically (Figure 4e; dark blue bars) and the cellular DNA content also increased (Figure 4f; dark blue bars). This demonstrates that additional DnaA triggers excessive replication initiation as also previously observed (Atlung *et al*., 1987; Skarstad *et al*., 1989).

When DnaA was overproduced in cells with high ppGpp that otherwise led to cessation of initiation, we observed that initiation of replication continued as the number of cellular origins increased and replication failed to complete in the presence of rifampicin (Figure 4d). The *ori/ter* ratio remained high (Figure 4e; light blue bars) and the cellular DNA content increased (Figure 4f; light blue bars) despite of a high ppGpp level (Figure 3c). The increase in DNA content resulted from continued initiation and elongation of replication in cells that were severely growth‐impaired and hence non‐dividing.

Overall, these data show that continued de novo DnaA protein synthesis can overcome the inhibition of chromosome replication initiation caused by an increasing ppGpp level.

### 2.3 Transcriptional reprograming contributes to arrest initiation of chromosome replication at elevated ppGpp levels

The ability of DnaA overproduction to stimulate replication initiation was somewhat dampened by a concurrent increase in ppGpp level as seen by the less dramatic increase in both *ori/ter* ratios and cellular DNA content observed for cells overproducing DnaA during ppGpp accumulation as compared to wild‐type cells overproducing DnaA (Figure 4c‐f). This indicates that high levels of ppGpp also can lower replication initiation by mechanisms independent of DnaA.

To address additional role(s) of ppGpp on replication initiation ppGpp synthesis was induced in cells containing a mutant RNAP that is non‐responsive, that is, does not bind ppGpp due to several amino acid substitutions in RpoC as well as deletion of amino acids 2‐5 in RpoZ (referred to as −1−2 (Ross *et al*., 2016)). This allowed the clarification of whether the effect of ppGpp on replication initiation was mediated through RNAP‐driven transcriptional changes only, or whether there is an additional direct effect of ppGpp on replication proteins.

**FIGURE 5:**
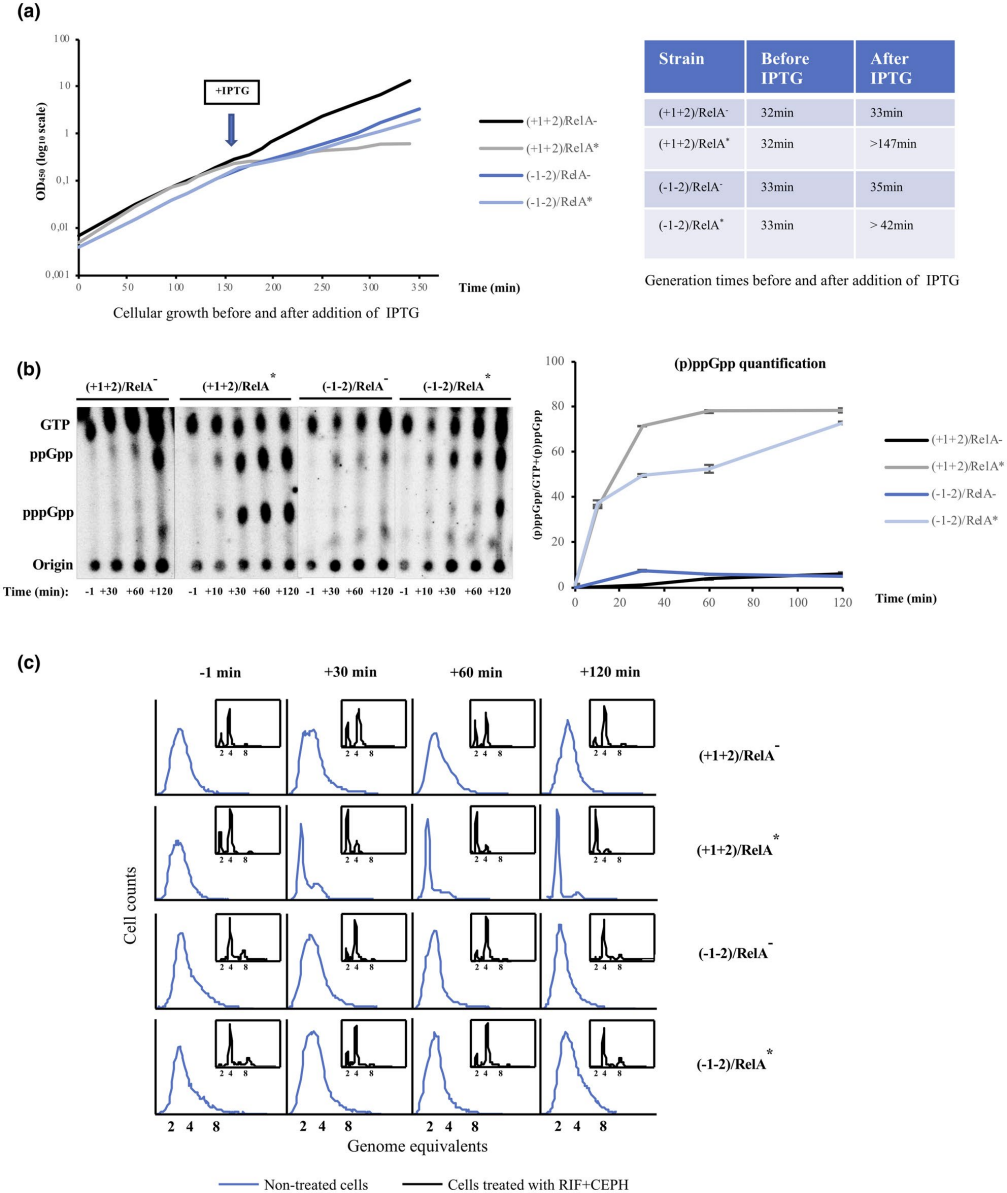
The effect of ppGpp on replication initiation is mediated through RNAP‐driven transcriptional changes. MG1655 cells with either the wild‐type RNAP (+1+2; ALO5045) or the ppGpp‐insensitive mutant RNAP (−1−2; ALO6798) carrying either the “control” plasmid with the *relA* ^–^ gene expressed downstream the p_A1/04‐03_ promoter, pBR322‐RelA^–^ (relA^–^), or the RelA expression plasmid with the relA^*^ gene expressed downstream the p_A1/04‐03_ promoter, pBR322‐RelA^*^ (relA^*^), were grown at 37°C in AB minimal medium supplemented with glucose and casamino acids. At time 0 min 1 mM IPTG was added to induce RelA synthesis. (a) Growth curves for +1+2 cells carrying pBR322‐RelA^–^ (black) or pBR322‐RelA^*^ (gray), and for −1−2 cells carrying pBR322‐RelA^–^ (dark blue) or pBR322‐RelA^*^ (light blue). Optical density at 450nm (OD_450_) is depicted as a function of time in a semilogarithmic diagram. Time of addition of 1 mM IPTG is indicated. Generation times before and after IPTG addition are indicated in the table. (b) Quantification of (p)ppGpp accumulation (total amount of ppGpp and pppGpp) in +1+2 and −1−2 cells carrying either pBR322‐RelA^–^ or pBR322‐RelA^*^ at the given times. Autoradiograms were used to calculate and visualize the fractional content of (p)ppGpp as a function of time. Standard deviations are based on three technical replicates. (c) Samples of exponentially growing +1+2 and −1−2 cells carrying either pBR322‐RelA^–^ or pBR322‐RelA^*^ were removed at the given times for direct analysis by flow cytometry (marked by blue). Samples of exponentially growing +1+2 and −1−2 cells carrying either pBR322‐RelA^–^ or pBR322‐RelA^*^ were removed at the given times for treatment with rifampicin and cephalexin prior to flow cytometry analysis (inserts).

Induction of RelA^*^ synthesis in wild‐type cells slowed down growth significantly, most likely due to ppGpp accumulation, whereas growth of the −1−2 strain was largely unaffected by an increase in ppGpp level (Figure 5a,b). Accumulation of ppGpp resulted in cessation of replication initiation leading to complete run out replication over time in wild type (+1+2), but not in −1−2, cells (Figure 5c). Further, rifampicin and cephalexin treatment of −1−2 cells showed that ppGpp accumulation neither affected the cellular number of replication origins nor the synchrony of initiation to any significant level (Figure 5c; insert). Therefore, the inhibiting effect of ppGpp on DNA replication initiation is mediated indirectly through RNAP‐driven reprograming of transcription and not through altering the activity of replication proteins. Thus, a change in one or more ppGpp‐regulated gene transcripts works in concert with a lowered DnaA level to limit replication initiation during ppGpp accumulation.

### 2.4 Chromosome replication initiation is more sensitive to transcriptional than translational inhibition

We proceeded to test whether the efficient turn‐off of replication initiation imposed from rifampicin resulted from its effect on protein, that is, DnaA, synthesis only, or whether the inhibitory effect was enhanced by inhibition of additional transcripts as well. The kinetics of replication initiation inhibition of rifampicin was compared to that of chloramphenicol; a bacteriostatic and broad‐spectrum antibiotic that acts on the 50S ribosomal subunit to inhibit translation without affecting transcription (Vazquez, 1979).

Previously, rifampicin and chloramphenicol have been shown to shut down transcription and translation, respectively, within 5–10 s after addition to highly permeable AS19 cells (Pato and Von Meyenburg, 1970; Pato *et al*., 1973).

**FIGURE 6:**
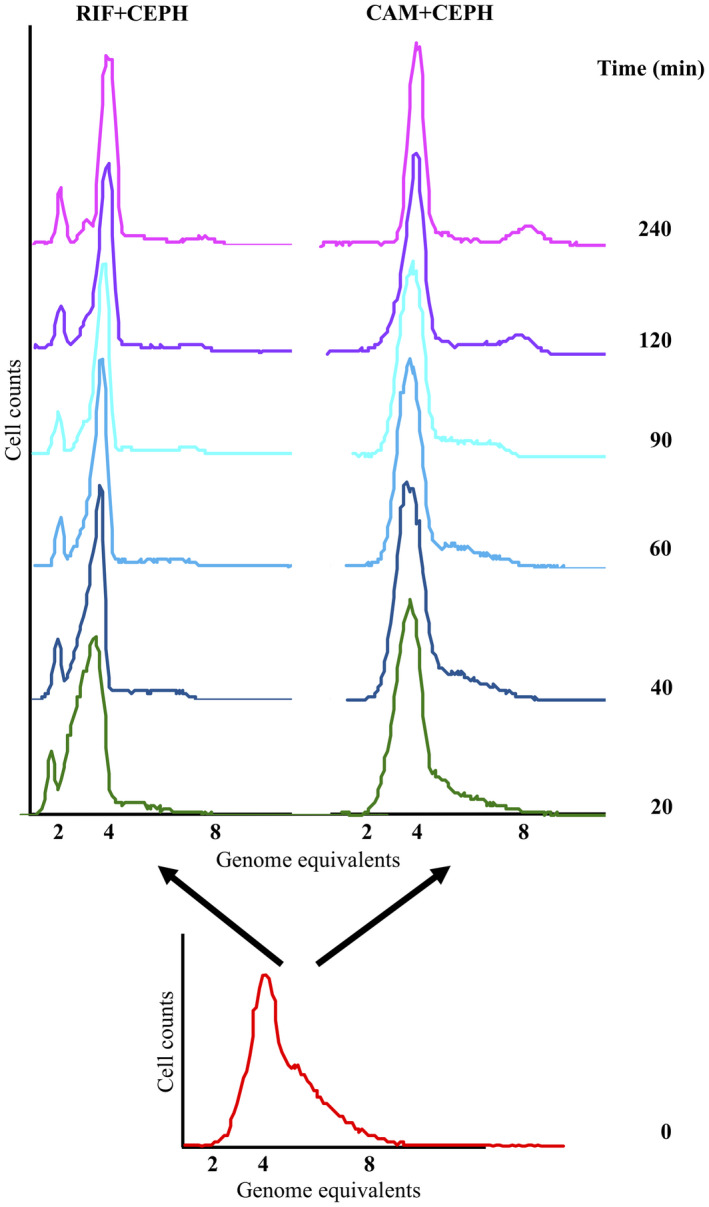
Inhibition of transcription inhibits replication initiation faster than inhibition of translation. *E. coli* AS19 cells were grown exponentially at 37°C in AB minimal medium supplemented with glucose and casamino acids. At time 0 min the culture was split in two. One subculture received rifampicin and cephalexin (“RIF + CEPH,” left) and one received chloramphenicol and cephalexin (“CAM + CEPH,” right) to inhibit initiation of RNA synthesis and cell division, or protein synthesis and cell division, respectively. Samples for flow cytometry analysis were removed at the indicated times. Samples representing a complete replication run out were removed 4 hr after drug addition

Rifampicin and cephalexin or chloramphenicol and cephalexin was added to exponentially growing cultures of AS19 cells, respectively, and the residual DNA synthesis was followed over time (Figure 6). Because cephalexin inhibits cell division, the number of fully replicated chromosomes per cell following run out synthesis will depend on how fast each antibiotic inhibited replication initiation, whereas the time required to finish replication will depend on how the replication elongation rate is affected. Cells treated with rifampicin and cephalexin finished replication approximately 60–90 min after drug addition, and contained mainly two, four, and eight fully replicated chromosomes with an average of 4.1 cellular origins (Figure 6; left). Cells treated with chloramphenicol and cephalexin ended up with primarily four and eight fully replicated chromosomes, and the average number of cellular origins was 5.1, that is, higher than for rifampicin treated cells (Figure 6; right), which indicates that rifampicin inhibits replication initiation in a faster mode than chloramphenicol. Chloramphenicol treated cells took longer (≥120 min) to finish replication most likely due to chromatin condensation (Zimmerman, 2002), hence slowing down the chromosome replication rate. Similar results were obtained with MG1655 (Supporting Information Figure S1).

Accumulation of DnaA ceases immediately in chloramphenicol treated cells due to inhibition of translation and somewhat later in rifampicin treated cells due to the residual capacity for synthesis from the *dnaA* mRNA present at the time of rifampicin addition. Yet, chloramphenicol treated cells continued initiation for a longer period relative to rifampicin treated cells. As transcription is still on‐going in chloramphenicol treated cells, this suggests that the efficient inhibition of chromosome replication initiation seen for rifampicin treated cells is caused via both a stop in de novo protein, that is, DnaA synthesis, and an arrest of transcription. This also explains why overinitiation caused by T7 RNAP‐directed DnaA synthesis was lowered in cells treated with rifampicin relative to untreated cells where transcription continues (compare Figures 2 and 4).

## 3 DISCUSSION

In *E. coli*, rifampicin‐mediated inhibition of transcription and altered global transcription triggered by high ppGpp levels result in an arrest in replication initiation. Both treatments reduce *dnaA* gene transcription and hence reduce de novo DnaA protein synthesis. Here, we show that continued DnaA synthesis allows for replication initiation both in the presence of rifampicin and during ppGpp accumulation, indicating that both rifampicin and high ppGpp halt initiation through reduced de novo DnaA protein synthesis. The effect of DnaA overproduction during rifampicin treatment or high ppGpp was, however, dampened compared to untreated/wild‐type cells. This indicates that reduced/altered transcription of genes in addition to *dnaA* contribute to initiation inhibition.

### 3.1 Replication initiation and de novo DnaA synthesis

In *E. coli*, initiation of chromosome replication is inhibited during rifampicin treatment (von Meyenburg *et al*., 1979), which blocks initiation of global RNA synthesis through direct binding to‐ and inhibition of RNAP. Here, we found that de novo synthesis of DnaA from the rifampicin‐insensitive T7 RNAP‐dependent p_T7_ promoter was sufficient to continue replication initiation in the presence of rifampicin. This is in agreement with an earlier report where high levels of DnaA allowed for some additional initiations from *oriC* in the presence of rifampicin (Pierucci *et al*., 1987). This underlines the importance of de novo DnaA protein synthesis for initiation and suggests that rifampicin inhibits this process by preventing *dnaA* gene transcription and hence de novo DnaA protein synthesis.

While accumulation of ppGpp in wild‐type cells resulted in initiation inhibition (Levine *et al*., 1991; Ferullo and Lovett, 2008), this was not the case for cells carrying a mutated RNAP non‐responsive to ppGpp (i.e., −1−2; (Ross *et al*., 2016)). Therefore, ppGpp imposed changes in RNAP‐driven transcription of one or several genes are responsible for inhibition of initiation, whereas there is no evidence for a direct ppGpp effect on proteins involved in replication initiation as also reported previously (Kraemer *et al*., 2019). This is in line with findings that DnaA is not a direct target for ppGpp (Zhang *et al*., 2018; Wang *et al*., 2019). de novo DnaA protein synthesis directed from the ppGpp‐insensitive T7 RNAP‐dependent p_T7_ promoter was sufficient to sustain continued replication initiation during ppGpp accumulation. Therefore downregulation of *dnaA* gene transcription (Chiaramello and Zyskind, 1990) and reduced de novo DnaA protein synthesis is responsible for replication initiation inhibition by ppGpp in wild‐type cells. We suggest that high levels of ppGpp inhibit replication initiation in a manner similar to rifampicin, that is, mainly by halting de novo DnaA protein synthesis.

### 3.2 Importance of DnaA activity

Recently it was found that overproduction of DnaA induced overinitiation in *E. coli,* but subsequent induction of ppGpp synthesis still inhibited replication initiation under these conditions (Kraemer *et al*., 2019). This would seemingly contradict the present finding that continued de novo DnaA synthesis allows for new rounds of replication initiation during accumulation of ppGpp and during rifampicin treatment. A major difference between the two experimental setups is that in the previous report (Kraemer *et al*., 2019), DnaA was overproduced prior to ppGpp accumulation, while it is overproduced during ppGpp accumulation in the data presented here. Overproduction of DnaA in wild‐type cells induces a burst in initiations (Atlung *et al*., 1987; Skarstad *et al*., 1989), which can be explained by de novo DnaA protein being mainly ATP bound and thus active. This is due to ATP being more abundant than ADP within the cell (Petersen and Møller, 2000), and because DnaA binds these nucleotides with similar affinity (Sekimizu *et al*., 1987). These initiations accelerate processes that convert DnaA^ATP^ to DnaA^ADP^, and a new steady state is reached where replication initiation takes place at a slightly reduced mass, and without any significant increase in the DnaA^ATP^‐to‐DnaA^ADP^ ratio (Flatten *et al*., 2015); that is, the activity of DnaA for initiation action is unchanged or only moderately increased. Moreover, overproduction of mainly ATP‐bound DnaA in RIDA deficient cells leads to massive overinitiation and inviability (Riber and Lobner‐Olesen, 2005; Fujimitsu *et al*., 2008). Based on these observations, we suggest that it is primarily the activity of DnaA, that is, DnaA^ATP^‐to‐DnaA^ADP^ ratio, which determines whether or how fast initiations cease in response to rifampicin treatment or an elevated ppGpp level, and that both conditions fail to arrest replication initiation when this ratio is high. In agreement with this, both rifampicin treatment and ppGpp fail to arrest initiations in cells that overproduce a hyperactive DnaA protein that mimics DnaA in its ATP bound form (Simmons *et al*., 2004, Kraemer *et al*., 2019).

When DnaA is de novo synthesized in the presence of either high ppGpp or rifampicin as presented here, the pool of DnaA^ATP^ is continuously replenished and the cellular DnaA^ATP^‐to‐DnaA^ADP^ ratio increased. An increased ATP level during the stringent response and during rifampicin treatment (Lobritz *et al*., 2015; Varik *et al*., 2017) could add to the increase. As the level of T7‐mediated DnaA overproduction was quite high it may overpower the ability of RIDA and DDAH to reduce the DnaA^ATP^‐to‐DnaA^ADP^ ratio, which could even further add to increasing the overall DnaA^ATP^‐to‐DnaA^ADP^ ratio.

Consequently, cells with continued de novo DnaA synthesis are able to continue initiation, at least for a while, both during ppGpp accumulation and during rifampicin treatment.

These observations underline the importance of DnaA activity and indicate that initiation inhibition by ppGpp accumulation or rifampicin treatment results, at least in part, from failure to replenish the DnaA^ATP^ pool.

### 3.3 Transcriptional activation of the origin

Recently, it was suggested that ppGpp inhibits replication initiation mainly by reducing the negative superhelicity of *oriC*, either by reduced transcription of the *mioC* and *gidA* genes adjacent to *oriC* (Kraemer *et al*., 2019), or by reducing gyrase and topoisomerase IV expression (Fernandez‐Coll *et al*., 2020). Transcriptional activation is, however, not a requirement for replication initiation in wild‐type cells, and both *mioC* and *gidA* promoters can be deleted without measurable effects (Lobner‐Olesen and Boye, 1992; Bates *et al*., 1997; Lies *et al*., 2015). Moreover, transcriptional activation becomes important for initiation during suboptimal conditions, such as when the chromosomal *oriC* is truncated by deletion of DnaA binding site R4 (Bates *et al*., 1997).

Here we show that accumulation of ppGpp prevents replenishment of the DnaA pool, presumably by reducing *dnaA* gene transcription. DnaA therefore becomes limiting for initiations. We suggest that transcriptional activation of *oriC* can assist during these suboptimal conditions and delay the replication initiation arrest. This explains why replacement of the ppGpp regulated *gidA* promoter (Sanchez‐Vazquez *et al*., 2019) with the ppGpp‐independent p_T7_ promoter allowed cells to continue initiations for a while during ppGpp accumulation (Kraemer *et al*., 2019) when the DnaA level/activity is suboptimal for initiation. The negative effect of ppGpp on the *gidA* promoter (Sanchez‐Vazquez *et al*., 2019) may also explain why we observe that overinitiation resulting from DnaA overproduction is dampened by a high ppGpp level.

The same applies to rifampicin that negatively affects the *gidA* promoter as well. In rifampicin treated cells, accumulation of DnaA gradually ceases due to the lack of transcription, whereas translation continues as long as intact *dnaA* mRNA is present. The translational inhibitor chloramphenicol, moreover, arrests DnaA accumulation immediately. Yet, we found rifampicin treatment to stop replication initiation faster than chloramphenicol. This is in line with previous findings that RNA synthesis contributes directly to an early stage of chromosome replication initiation (Lark, 1972; Messer, 1972; Zyskind *et al*., 1977). As chloramphenicol enables continued transcription, this supports that transcriptional activation of the origin allows for, at least a few, additional initiations during suboptimal, that is, DnaA limiting, conditions, and that cessation of de novo DnaA synthesis along with an inability to transcriptionally activate *oriC,* leads to a faster replication initiation stop as seen for rifampicin treated cells.

Previously, a direct interaction of DnaA with RNAP at *oriC* was reported to partially protect RNA polymerase against rifampicin at the *gidA* promoter (Flatten *et al*., 2009). The increased DnaA levels reported here could therefore allow for a degree of continued transcriptional activation mediated by *gidA* transcription in the presence of rifampicin, which in turn could contribute to continued replication initiation in the presence of rifampicin.

In summary, we propose that binding of rifampicin or ppGpp to RNAP leads to a stop/reduction in de novo DnaA protein synthesis. Although this results in a failure to replenish the DnaA^ATP^ pool, which should be sufficient for an arrest in replication initiation, it does not ensure a fast turn‐off. The efficacy of both rifampicin and ppGpp in turning off initiations is further enhanced by their negative effect on transcriptional activation of *oriC*. Therefore, a combination of several mechanisms including reduced de novo DnaA synthesis and lower negative supercoiling of *oriC* resulting from either reduced *gidA* (Kraemer *et al*., 2019) or *gyrA/parC* (Fernandez‐Coll *et al*., 2020) transcription contributes to efficiently shutting down initiations during ppGpp accumulation or rifampicin treatment.

## 4 EXPERIMENTAL PROCEDURES

### 4.1 Media and growth conditions

Cells were grown at 37°C in AB minimal medium supplemented with 0.2% glucose, 0.5% casamino acids, and 10 μg/ml thiamine. When necessary, antibiotics were added at the following concentrations: kanamycin, 50 μg/ml; chloramphenicol, 20 μg/ml; tetracycline, 10 μg/ml; carbenicillin, 200 μg/ml.

### 4.2 Bacterial strains

Bacterial background strains are listed in Table 1.

**TABLE 1:** Bacterial strains used in this study

Strain	Genotype	Reference/Source
MG1655	F^–^ λ^–^ *rph‐1*	(Guyer *et al*., 1981)
ALO6511	λDE3 *rph^+^, gatC^+^, glpR^+^* ^a^	(Tseng *et al*., 2009)/This work
ALO5045	*rpoZ‐kan^R^, rpoC‐tetA^R^* (+1+2)^a^, ^b^	(Ross *et al*., 2016)/This work
ALO6798	*rpoZ* Δ(2‐5), *rpoC* R362A R417A K615A N680A K681A (−1−2)^a^	(Ross *et al*., 2016)/This work
AS19	Complex. For genome sequence see (Avalos *et al*., 2018)	(Sekiguchi and Iida, 1967)

^a^ Genotype otherwise as MG1655.

^b^ WT *rpoZ* with kan‐cassette inserted between *dinD* and *yicG*. WT *rpoC* with tetA‐cassette inserted between *rpoC* and *yjaZ*.

### 4.3 Construction of bacterial plasmids

All plasmids are listed in Table 2. Primers are listed in Table 3.

**TABLE 2:** Plasmids used in this study

Plasmid	Relevant genotype	Relevant phenotype	Reference/Source
pBR322	*bla, tet*	None	Bolivar *et al*. (1977)
pFH2102	*ori*‐pBR322, *lacP_A1/04‐03_, lacI, bla*	None	von Freiesleben *et al*. (2000)
pBR322‐RelA^*^	*ori*‐pBR322, lacP_A1/04‐03‐_relA^*^, lacI, bla	Functional RelA; accumulation of ppGpp upon induction	This work
pBR322‐RelA^–^	*ori*‐pBR322, *lacP_A1/04‐03‐_relA* ^–^ *, lacI, bla*	Inactive RelA; “empty”	This work
pET26b(+)	*ori*‐pBR322, *P_T7_*, C‐terminal His_6_‐tag, *kana*	T7 expression vector	Novagen, EMD Millipore
pACYC184	*ori*‐p15A, *tet, cat*	None	Chang and Cohen (1978)
pACYC184‐T7‐DnaA	*ori*‐p15A, *P_T7_‐dnaA, tet*	Expression of DnaA upon induction	This work
pACYC184‐T7	*ori*‐p15A, *P_T7_, tet*	None; “empty”	This work

**TABLE 3:** Oligonucleotides used in this study

Name	Sequence
relA_up_XbaI_bw	5ʹ‐TCGAGCTCTAGATTAAGGAGGCCATATGGTTGCGGTAAGAAGTGCAC
relA_down13_BamHI_fw	5ʹ‐ GATATCGGATCCCTTCTCTCATCCGCCAAAACAGC
relA_down14_BamHI_fw	5ʹ‐ GATATCGGATCCCTGTATCAGGCTGAAAATCTTCTC
DnaA_539_fw_XbaI	5ʹ‐ GGCGGCTCTAGACGACGTACGTCAACAATCATG
DnaA_539_bw_XhoI	5ʹ‐ GGCGGCCTCGAGGGACCGCTCACCTGTTGTAGC
T7_dnaA_bw_NcoI	5ʹ‐ GGCGGCCCATGGCGCCAATCCGGATATAGTTCC
T7_26b_dnaA_fw_NcoI	5ʹ‐ GGCGGCCCATGGGTGATGTCGGCGATATAGGC

Plasmid pBR322‐RelA^*^ encodes a functional RelA^*^ protein and was constructed by amplifying a 1,440 bp PCR fragment encoding the 455 N‐terminal amino acids of plasmid pALS13 (Svitil *et al*., 1993) using primers relA_up_XbaI_bw and relA_down13_BamHI_fw. A corresponding “empty” plasmid, pBR322‐RelA^–^, was generated by amplifying a 1,086 bp PCR fragment encoding the 331 N‐terminal amino acids of plasmid pALS14 (Svitil *et al*., 1993) using primers relA_up_XbaI_bw and relA_down14_BamHI_fw. Both PCR fragments were digested with BamHI and XbaI and ligated into plasmid pFH2102 (von Freiesleben *et al*., 2000) downstream of the IPTG inducible p_A1/04‐03_ promoter (Lanzer and Bujard, 1988), cut with the same enzymes, to generate plasmids pBR322‐RelA^*^ and pBR322‐RelA^–^, respectively.

To construct a plasmid expressing DnaA in a rifampicin‐ and ppGpp‐independent manner, the *dnaA* gene with ribosome binding site (RBS) was amplified as a 1,560 bp PCR fragment from MG1655 using primers DnaA_539_fw_XbaI and DnaA_539_bw_XhoI. This fragment was digested with XbaI and XhoI and ligated into the expression vector, pET26b(+) (Novagen, EMD Millipore, WI, US), cut with the same enzymes, resulting in pET26b‐DnaA. To generate plasmid pACYC184‐T7‐DnaA, a 1,880 bp fragment carrying the *dnaA* gene downstream of the T7 RNAP‐dependent promoter of pET26b(+), p_T7_, was PCR amplified from pET26b‐DnaA using primers T7_dnaA_bw_NcoI and T7_26b_dnaA_fw_NcoI. The fragment was digested with NcoI and ligated into the *cat* gene of pACYC184 cut with the same enzyme. The corresponding “empty” plasmid, pACYC184‐T7, was constructed by amplifying a 480 bp PCR fragment directly from the vector pET26b(+) carrying only the T7 RNAP‐dependent promoter, p_T7_, and the multicloning site (MCS) using the primers T7_dnaA_bw_NcoI and T7_26b_dnaA_fw_NcoI. The resultant fragment was digested with NcoI and ligated into the *cat* gene of pACYC184.

### 4.4 Flow cytometry and cell cycle analysis

Prior to flow cytometry analysis, exponentially growing cells (OD_450_ = 0.1–0.3) were treated with rifampicin and cephalexin to inhibit initiation of RNA synthesis and cell division, respectively (Lobner‐Olesen *et al*., 1989), or chloramphenicol and cephalexin to inhibit protein synthesis and cell division, respectively (Skarstad *et al*., 1986). Drugs were used at the following final concentrations: rifampicin, 300 μg/ml (SERVA Electrophoresis GmbH); chloramphenicol, 200 μg/ml (Sigma‐Aldrich); cephalexin, 36 μg/ml (Sigma‐Aldrich). Incubation continued for a minimum of 4 hr at 37°C, unless otherwise specified. When replication initiation is inhibited, and ongoing rounds of replication allowed to finish, the number of fully replicated chromosomes per cell (i.e., genome equivalents) represents the number of origins per cell at the time of drug addition. In case of initiation synchrony, the integral number of chromosomes is two, four, or eight (i.e., 2^n^). In cells with asynchronous initiation additional peaks appear representing three, five, six, or seven cellular origins (i.e., ≠2^n^). Flow cytometry was performed as described previously (Løbner‐Olesen *et al*., 1989) using an Apogee A10 Instrument (Apogee, Inc.). For each sample, 40,000–100,000 cells were analyzed.

### 4.5 Immunoblotting

Samples of 2 ml of exponentially growing cells (OD_450_ = 0.15–0.35) were harvested, and cell pellets were adjusted to the same total protein content using the Bio‐Rad Protein Assay Dye Reagent Concentrate Kit (Bio‐Rad, Laboratories, Inc., CA, US (Bradford, 1976). Samples of 2.2 to 2.6 μg of total protein were separated by 4%–12% SDS‐polyacrylamide gel electrophoresis. Detection of DnaA was carried out by semidry Western blotting using rabbit antiserum raised against DnaA protein followed by ECF fluorescence detection (GE Healthcare, IL, US) as previously described (Riber and Lobner‐Olesen, 2005). The membranes were scanned using a GenoView imaging system equipped with a UV transilluminator (VWR, PA, US). Quantification was done using the ImageJ software (LOCI, University of Wisconsin, US).

### 4.6 Quantification of cellular levels of (p)ppGpp

Cultures were grown in low phosphate (0.2 mM K_2_HPO_4_) MOPS minimal medium supplemented with 0.2% glucose, 0.5% casamino acids and 10 μg/ml thiamine to an OD_450_ of 0.150. Cultures were labeled with ^32^P (final specific activity 100 μCi/ml; Hartmann Analytic GmbH, Braunschweig, Germany) for 2–3 generations of growth. ppGpp accumulation was induced by the addition of 1 mM IPTG. At the times indicated, samples of 50 μl were removed and mixed with 10 μl of ice‐cold 2 M formic acid. Samples were kept at ice and stored at −20°C.

Prior to ppGpp detection, the frozen samples were centrifuged at 4°C for 45 min at 15,000*g* to pellet the cell debris, and 5 μl aliquots of the supernatants were added to polyethyleneimine (PEI) cellulose thin layer chromatography plates (Merck, Sigma, MO, US), and resolved with 1.5 M KH_2_PO_4_ (pH 3.4) before being air dried and exposed by phosphorimaging (Amersham Typhoon phosphorimager, GE Healthcare, IL, US). Quantification of cellular (p)ppGpp levels was done using the ImageJ software (LOCI, University of Wisconsin, US), and the relative abundance of ppGpp and pppGpp was measured as a percentage value relative to the total amount (ppGpp+pppGpp+GTP) as previously described (Mechold *et al*., 2013).

### 4.7 Quantitative polymerase chain reaction, qPCR

Cells fixed in 70% ethanol were collected by centrifugation of 200 μl aliquots at 4°C for 15 min at 15,000*g*. Cells were washed twice in the same volume of water in order to carefully remove all traces of ethanol before being applied directly as templates in the qPCR reactions. The qPCR was performed using SYBR Premix Ex Taq II (RR820A, Takara Bio Inc., Kusatsu, Japan) in a total volume of 20 μl with 2 μl of template (see above) and 0.4 μM of each primer. The following program was applied in a BioRAD CFX96 machine (Bio‐Rad, Laboratories, Inc., CA, US); 95°C for 30 s, 39 × (95°C for 5 s + 60°C for 30 s), 95°C for 15 s and finally 60°C for 60 s. Origins were quantified using primers that amplified part of the *gidA* gene (5ʹ‐TTCGATCACCCCTGCGTACA‐3ʹ and 5ʹ‐CGCAACAGCATGGCGATAAC‐3ʹ), whereas termini were detected using primers that amplified part of the *dcp* gene (5ʹ‐TTGAGCTGCGCCTCATCAAG‐3ʹ and 5ʹ‐TCAACGTGCGAGCGATGAAT‐3ʹ), as previously reported (Riber *et al*., 2006). All *ori/ter* ratios were normalized to the *ori/ter* ratio of a sample of wild‐type cells treated with rifampicin and cephalexin where the *ori/ter* ratio is one.

## AUTHOR CONTRIBUTIONS

LR and ALO have both made major contributions to (a) the conception or design of the study, (b) the acquisition, analysis, or interpretation of the data; and (c) writing of the manuscript.

## Supporting information

Fig S1

## Data Availability

The data that supports the findings of this study are available from the corresponding authors upon a reasonable request.
