# Supplementary material for: Inhibition of Escherichia coli chromosome replication by rifampicin treatment or during the stringent response is overcome by de novo DnaA protein synthesis
Source: Mol Microbiol. 2020 Jun 15;114(6):906–19. doi: 10.1111/mmi.14531 (PMC7818497; doi:10.1111/mmi.14531)
Supplement: Supplementary file 1 — Fig S1 [file MMI-114-906-s001.pdf]

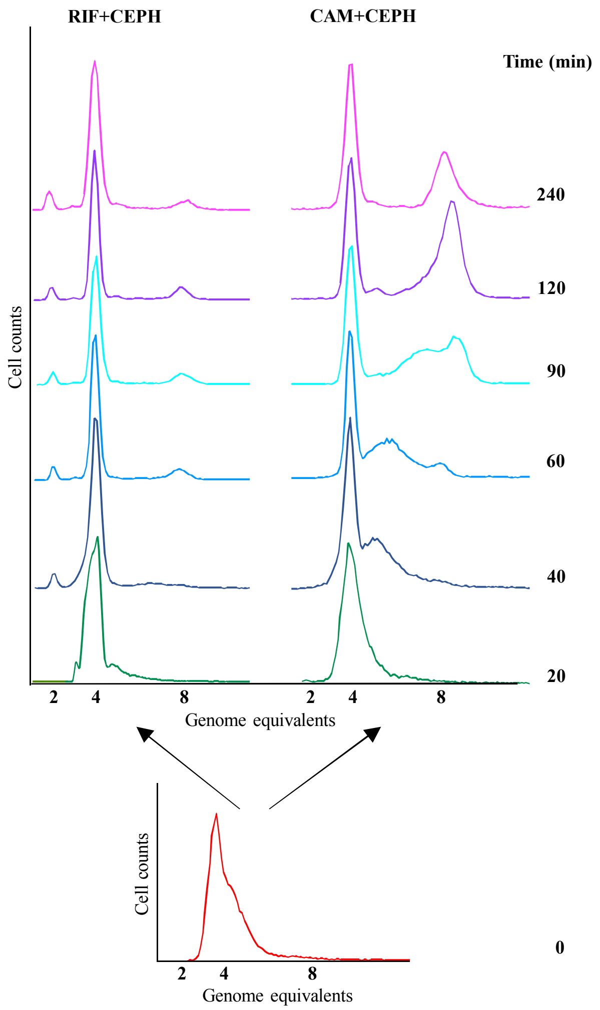

Supporting Figure S1. Inhibition of transcription inhibits replication initiation faster than inhibition of translation

MG1655 cells were grown exponentially at 37 °C in AB minimal medium supplemented with glucose and casamino acids. At time 0 minutes the culture was split in two. One subculture received rifampicin and cephalexin **("RIF+CEPH", left**) and one received ~~chloramphenicol~~ chloramphenicol and cephalexin **("CAM+CEPH", right**) to inhibit initiation of RNA synthesis and cell division, or protein synthesis and cell division, respectively. Samples for flow cytometry analysis were removed at the indicated times. Samples representing a complete replication runout were removed 4 hours after drug addition.
